# Direct observation of the thermal demagnetization of magnetic vortex structures in nonideal magnetite recorders

**DOI:** 10.1002/2016GL070074

**Published:** 2016-08-19

**Authors:** Trevor P. Almeida, Adrian R. Muxworthy, András Kovács, Wyn Williams, Leslei Nagy, Pádraig Ó Conbhuí, Cathrine Frandsen, Radchagrit Supakulopas, Rafal E. Dunin‐Borkowski

**Affiliations:** ^1^ Department of Earth Science and EngineeringImperial College London, South Kensington CampusLondonUK; ^2^ Now at School of Physics and Astronomy, Kelvin BuildingUniversity of GlasgowGlasgowUK; ^3^ Ernst Ruska‐Centre for Microscopy and Spectroscopy with Electrons and Peter Grünberg InstituteForschungszentrum JülichJülichGermany; ^4^ School of GeoSciencesUniversity of EdinburghEdinburghUK; ^5^ Department of PhysicsTechnical University of DenmarkKongens LyngbyDenmark

**Keywords:** magnetite, thermal demagnetization, paleomagnetism

## Abstract

The thermal demagnetization of pseudo‐single‐domain (PSD) magnetite (Fe_3_O_4_) particles, which govern the magnetic signal in many igneous rocks, is examined using off‐axis electron holography. Visualization of a vortex structure held by an individual Fe_3_O_4_ particle (~250 nm in diameter) during in situ heating is achieved through the construction and examination of magnetic‐induction maps. Stepwise demagnetization of the remanence‐induced Fe_3_O_4_ particle upon heating to above the Curie temperature, performed in a similar fashion to bulk thermal demagnetization measurements, revealed that its vortex state remains stable under heating close to its unblocking temperature and is recovered upon cooling with the same or reversed vorticity. Hence, the PSD Fe_3_O_4_ particle exhibits thermomagnetic behavior comparable to a single‐domain carrier, and thus, vortex states are considered reliable magnetic recorders for paleomagnetic investigations.

## Key Points

- Thermal demagnetization of nonideal magnetite particles
- Visualized magnetization of vortex state with temperature
- Vortex structures are reliable paleomagnetic signal recorders

## 1 Introduction

Paleomagnetists study the magnetic signal recorded by magnetic minerals within rocks to understand a wide range of geological problems, e.g., plate tectonic movements and geomagnetic field variation. To facilitate reliable paleomagnetic data interpretation, the fundamental magnetic recording and laboratory recovery mechanisms must be fully understood. Thermoremanent magnetization (TRM) is the primary mechanism of remanence acquisition within igneous rocks and is recorded by the magnetic minerals within these rocks during cooling from above the Curie temperature *T_C_* (~580°C for magnetite (Fe_3_O_4_) [*Dunlop and Özdemir*, 1997]). Primary magnetizations are commonly partially reheated, and secondary magnetizations are acquired. To identify and isolate the primary and secondary magnetizations in the laboratory, it is necessary to “stepwise” demagnetize samples by incrementally heating the samples to increasing temperatures. Understanding the stability of the recorded signals after exposure to repeated heating events over geological and laboratory time scales is crucial for obtaining significant intensity and directional information. One factor that greatly influences the stability of the acquired signal is particle size, as the crystalline volume of the magnetic recorder energetically favors certain magnetic structures [*Dunlop and Argyle*, 1991]. Theories for TRM have been used to explain the response and behavior of submicron, uniformly magnetized grains termed single domain (SD) [*Néel*, 1949, 1955]; however, larger magnetic grains that exhibit nonuniform magnetic states (multidomain (MD)) dominate the magnetic signature of most rocks. In igneous rocks, the magnetic signal is predominantly recovered from small MD grains, frequently called pseudo‐single domain (PSD), due to their SD‐like recording fidelity. For a long time there has been uncertainty about the magnetic recording fidelity of PSD grains, regularly cited as ~0.1–10 µm for Fe_3_O_4_ [*Dunlop and Özdemir*, 1997]; PSD structures can vary within this size range and, by implication, the stability of the recorded signal. A vortex structure is considered a typical PSD state in Fe_3_O_4_ grains ~0.1–1 µm, and the stray magnetic field from its core provides the paleodirectional and paleointensity information. Until recently, knowledge of the thermoremanent behavior of PSD vortex structures was informed by numerical models [*Thomson et al*., 1994; *Winklhofer et al*., 1997], but these models are often insufficient and require much improvement, e.g., inclusion of scaled thermal fluctuations and/or unconstrained calculations of energy barriers, in order to clarify the fine details of PSD‐vortex stability at temperature [*Muxworthy et al*., 2003]. Hence, our current theoretical understanding of thermally active PSD is limited, and knowledge of the magnetic stability of most planetary paleomagnetic signals is lacking.

Off‐axis electron holography is an advanced transmission electron microscopy (TEM) technique that provides nanoscale imaging of the magnetic induction of materials [*Dunin‐Borkowski et al*., 1998; *Kasama et al*., 2013]. The technique produces high‐resolution images of the magnetic structures in nanoparticles; in recent years this has been frequently used in mineral magnetism [*Harrison et al*., 2002; *Almeida et al*., 2014a, 2014b]. In situ heating within the TEM was first combined with electron holography in a preliminary study to investigate directly the thermomagnetic behavior of an individual PSD Fe_3_O_4_ particle [*Almeida et al*., 2014c]. However, this initial experiment presented a vortex structure that exhibited magnetic signal well above the *T_C_* at 700°C and hence created questions about the reliability of the temperature measurements. This feasibility study also revealed several issues as it did not take into account contributions from the changing mean inner potential (MIP) with temperature [*Kamilov et al*., 1975]; thermal expansion [*Manahan*, 1990]; and most predominantly, from electrostatic charging [*Beleggia and Pozzi*, 2010]. A more recent study solved these issues and revealed that the particle size of PSD Fe_3_O_4_ grains plays a critical role in the thermomagnetic stability of vortex structures at elevated temperatures but also showed that the original signal was usually recovered on cooling [*Almeida et al*., 2016]. However, this heating cycle did not provide details on the reproducibility of the magnetic state. In order to acquire reliable magnetic information from the relatively large Fe_3_O_4_ grains (100 nm–250 nm) during repeated heating events, thermally induced sample tilt and the associated diffraction contrast must also be taken into consideration. The use of direct‐detection cameras (DDCs) within the TEM have been shown to improve significantly the interference fringe visibility within electron holograms [*Chang et al*., 2016] and is considered to aid markedly in the recovery of magnetic signal from samples with in situ tilt‐induced diffraction contrast.

As a significant step forward in our understanding of localized magnetization in magnetic recorders as function of temperature, this paper addresses all previous challenges and makes use of a DDC to visualize the true thermoremanent behavior of the vortex state in an individual Fe_3_O_4_ particle as it is heated above the *T_C_*. We report here the first ever sequence of images for the systematic stepwise demagnetization of PSD‐vortex remanence similar to the corresponding bulk thermal demagnetization measurements routinely performed in paleomagnetic laboratories.

## 2 Experiment

A powder of Fe_3_O_4_ grains (~150–250 nm in diameter), produced by hydrothermal synthesis, was purchased from Nanostructured and Amorphous Materials, USA. In order to prepare for heating in situ within the TEM, the powder was dispersed ultrasonically in ethanol before being deposited onto EMheaterchips^™^, with small electron‐transparent regions of silicon nitride (SiN) film, and then placed into a DENSsolutions TEM heating holder. Off‐axis electron holograms were obtained in Lorentz mode under zero field conditions in a Titan 80–300 TEM (300 kV) using a Gatan K2 Summit high‐speed DDC (Ernst Ruska‐Centre for Microscopy and Spectroscopy with Electrons, Forschungszentrum Jülich, Germany). A voltage of 90 V was typically applied to the electron biprism, resulting in an interference fringe spacing of ~3.9 nm. The DDC was operated in linear mode, where charge carriers generated by impinging electrons were accumulated over 6 s and read out to produce the electron hologram. The samples were initially heated in the TEM to a temperature of 700°C, allowing the residual water to evaporate and to relieve any strain that was introduced during crystal synthesis. Imaging of the magnetization during in situ heating was achieved through performing three separate experiments: (1) opposing directions of magnetization were initially induced and inverted within the Fe_3_O_4_ particle in situ within the TEM at room temperature by tilting ±70° and switching the microscope objective lens on (applied magnetic field >1.5 T). The objective lens was then switched off and the sample tilted back to 0° for acquisition of the holograms in field‐free conditions (residual field <0.2 mT); the sample had been given a saturation isothermal remanent magnetization (SIRM) at room temperature. Electron holograms were also acquired with the sample magnetized in opposite directions so that the mean inner potential (MIP) addition to the phase shift could be separated from the addition induced by magnetism, as explained by *Dunin‐Borkowski et al*. [1998]. Electron holograms were subsequently recorded on heating from 100°C up to 600°C at 100°C steps, and again upon cooling, all under field‐free conditions. The heating and cooling rates of the single‐tilt DENSsolutions heating holder were set at 50°C/min using the DENSsolutions temperature control. (2) The particle was then heated in a fashion similar to a bulk thermal demagnetization experiment, where the particle was again initially saturated at 20°C through reversing the magnetization by tilting ±70° and switching the objective lens on. Electron holograms were then recorded during in situ heating from 20°C to each 100°C interval and cooling back to 20°C in a stepwise fashion up to 600°C, e.g., 20°C → 100°C  → 20°C → 200°C → 20°C → 300°… → 600°C → 20°C, all under field‐free conditions. (3) Heating experiment 1 was then repeated, but in this instance, the magnetization inversion was executed by switching the objective lens on at ±70° at every 100°C step in order to determine the MIP as a function of temperature. The MIP addition to the phase shift was then removed from the unwrapped overall phase shift, obtained separately at individual 100°C intervals during experiments 1 and 2. We then constructed magnetic‐induction maps representative of the remanent magnetization. To create the representative magnetic‐induction maps, it is necessary to multiply the cosine of the magnetic addition to the phase to create contours of magnetic phase. The contours were colored to signify the projected induction direction, as depicted by color wheels (see below).

For comparison with standard paleomagnetic measurements, bulk magnetic measurements on the Fe_3_O_4_ powder were performed at the Natural Magnetism Group Laboratory (Imperial College). Thermal demagnetization procedures were carried out under flowing He in an ASC TD48 paleomagnetic oven, and measurements were made at room temperature following 20 demagnetization steps between 100°C and 600°C, reducing step sizes from 50°C to 25, 20 and 10°C with increasing temperature. For phase characterization of the Fe_3_O_4_ powder, a ^57^Fe Mössbauer spectrum was acquired at room temperature at the Technical University of Denmark, with the Mössbauer spectrum calibrated relative to α‐Fe.

Micromagnetic modeling of cuboctahedral geometries approximating the samples was performed using the finite element method. In this technique the magnetization, *m*, of a particle is calculated by minimizing the total free energy given by

$$E_{t}=\int_{\Omega }\left\{A{\left|\nabla m\right|}^{2}+K_{1}\left(m_{x}^{2}m_{y}^{2}+m_{x}^{2}m_{z}^{2}+m_{y}^{2}m_{z}^{2}\right)-M_{s}\left(H_{z}\cdot m\right)-\frac{1}{2}\left(H_{d}\cdot m\right)\right\}dV\text{,}$$

where *A* is the exchange constant, *K*_1_ is the magnetocrystalline anisotropy, *M_s_* is the saturation magnetization, ***H**_z_* is the applied field, and *H_d_* is the demagnetizing field. For each configuration of *m*, the gradient of the free energy *E_t_* is used to calculate a new value of *m* by a modified gradient descent method. When the change in energy is zero (i.e., suitably small on a computer), then the optimum energy configuration for the magnetization is reached.

A simulation of the magnetic‐induction map for a given micromagnetic model was performed by approximating the magnetization with a set of uniformly magnetized axis aligned bricks (UMAABs) and summing together the electron phase shift produced by each UMAAB at each pixel of the image. Closed, real‐space solutions for the magnetic addition to the phase shift due to each UMAAB are known [*Keimpema et al*., 2006], and the phase shift at a given pixel due to a set of UMAABs can be found by simply summing the phase shift at that pixel for each UMAAB. The magnetic‐induction map can then be found by taking the cosine of the phase shift, applying an amplification factor to produce magnetic phase contours, and coloring as previously described.

## 3 Results

**Figure 1:**
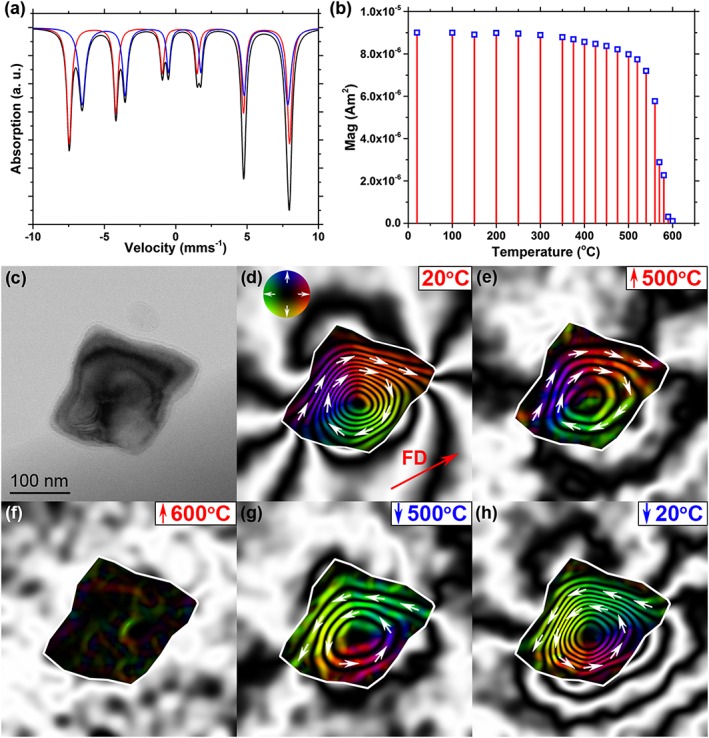
General crystallographic phase information and thermal demagnetization of Fe_3_O_4_ particles. (a) Mössbauer spectrum of the Fe_3_O_4_ powder, with two fine‐tuned sextets *St*_1_ (red) and *St*_2_ (blue). (b) Bulk thermal demagnetization measurement of the Fe_3_O_4_ powder acquired in the paleomagnetic laboratory at room temperature, following heating to increasing demagnetization temperatures. (c) BF TEM image of an individual Fe_3_O_4_ particle (major axis ~250 nm in length). Magnetic‐induction maps produced at (d) 20°C (the arrow shows the field direction (FD) of the applied SIRM), during in situ heating to (e) 500°C and (f) 600°C, as well as upon cooling to (g) 500°C and (h) 20°C. All five magnetic‐induction maps have the same contour spacing (0.53 radians). The direction of the magnetization is shown using arrows and the color wheel.

Figure 1 presents the magnetic properties of the Fe_3_O_4_ powder, as well as information on the morphology, grain size, and localized magnetization of an individual Fe_3_O_4_ particle.

The Mössbauer spectrum of Figure 1a provides crystallographic phase characterization of the oxidation state of the native Fe_3_O_4_ grains. The Mössbauer criteria were calculated by fine‐tuning two sextets, *St*_1_ (red) and *St*_2_ (blue), with region ratios of Lorentzian‐shaped lines limited to 3:2:1:1:2:3 in each case (Table 1). The two sextets in Figure 1a display hyperfine parameters that are indicative of pure Fe_3_O_4_. However, the weighted mean isomer shift of 0.46 mm/s (cf. *δ*_aver_ = 0.55 mm/s in pure Fe_3_O_4_) and the proportional spectral region of *St*_1_/*St*_2_ of 0.93 (cf. *St*_1_/*St*_2_ = 1.9 in pure Fe_3_O_4_ [*Da Costa et al*., 2014]) suggest only near‐stoichiometric Fe_3_O_4_. Since Fe_3_O_4_ readily oxidizes at ambient atmosphere and temperature conditions, and changes the spectral absorption pattern in a nontrivial way [*Da Costa et al*., 2014], the sample oxidation state was calculated as the percentage weight (wt%) of Fe_3_O_4_ in a Fe_3_O_4_/γ‐Fe_2_O_3_ (maghemite) mixture, by allowing for the weighted mean isomer shift *δ*_aver_ of *St*_1_ and *St*_2_ (Table 1), in addition to utilizing the linear interrelationship of *Da Costa et al*. [2014]:

$$\delta_{\text{aver}}\left(\mathrm{mm}/s\right)\;=\;0.335\;+\;0.00215\;\times \;Fe_{3}O_{4}\left(\mathrm{wt}\%\right)$$

**Table 1:** Mössbauer Parameters of Sextets *St*_1_ and *St*_2_ of Figure 1a Determined for the Fe_3_O_4_ Sample^a^

*B*_hf_ (*T*)		IS (mm s^−1^)		QS (mm s^−1^)		*W* (mm s^−1^)		*δ*_aver_	Fe_3_O_4_ (wt%)
*St*_1_	*St*_2_	*St*_1_	*St*_2_	*St*_1_	*St*_2_	*St*_1_	*St*_2_		
47.9	44.7	0.28	0.65	−0.01	0.01	0.39	0.55	0.46	59

a The columns are the magnetic hyperfine field (*B*_hf_), the isomer shift relative to metallic iron (IS), quadrupole splitting (QS), and the line width (*W*) of the most exterior lines of a sextet (FWHM), and *δ*_aver_ is the weighted mean isomer shift of *St*_1_ and *St*_2_. Fe_3_O_4_ following *Da Costa et al*. [2014].

Using the model of *Da Costa et al*. [2014] and assuming that all sample material is Fe_3_O_4_ and γ‐Fe_2_O_3_, a Fe_3_O_4_ content of 59 wt% is obtained. The bulk thermal demagnetization measurement of Figure 1b, to which the electron holography data will be compared, is in good agreement with that of PSD Fe_3_O_4_ [*Biggin et al*., 2013].

The bright‐field (BF) TEM image of Figure 1c reveals the Fe_3_O_4_ particle to exhibit an approximately rhombohedral shape in two‐dimensional projection, with a major axis of ~250 nm. The magnetic‐induction map of Figure 1d reveals its room temperature magnetization to take the form of a large clockwise spiraling vortex with dipole‐like stray magnetic field external to the grain, acting as good example of a PSD/vortex structure. Increasing the temperature to 500°C caused the magnetic contours to broaden due to decreasing intensity (Figure 1e), until heating to 600°C results in complete demagnetization (Figure 1f). The vortex PSD state is observed to be recovered when cooling back to 500°C (Figure 1g), and its magnetic intensity increases upon cooling to 20°C (Figure 1h), measured as ~95% the magnitude of the original vortex structure (Figure 1c). However, it is evident that the vorticity of the vortex structure is flowing in the opposite counterclockwise direction after the demagnetization process.

**Figure 2:**
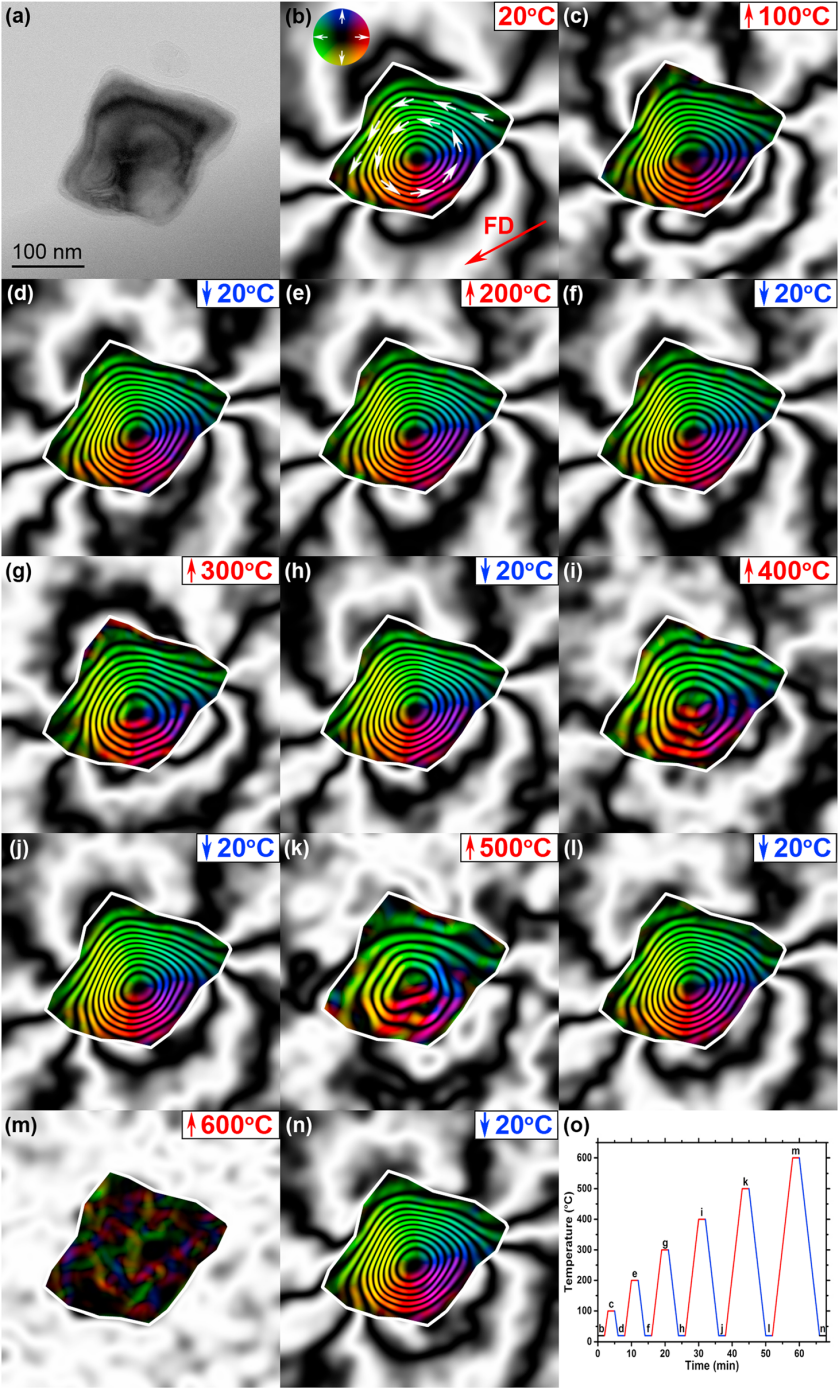
Visualization of the stepwise thermal demagnetization of an individual Fe_3_O_4_ particle. (a) BF TEM image of the Fe_3_O_4_ particle. Magnetic‐induction maps produced from electron holograms acquired at (b) 20°C (the arrow shows the field direction (FD) of the applied SIRM) and during in situ heating and cooling to (c) 100°C, (d) 20°C, (e) 200°C, (f) 20°C, (g) 300°C, (h) 20°C, (i) 400°C, (j) 20°C, (k) 500°C, (l) 20°C, (m) 600°C, and (n) 20°C. All the magnetic‐induction maps have the same contour spacing (0.53 radians). (o) Plot of the heating profile of the Fe_3_O_4_ particle during the stepwise thermal magnetization in relation to Figures 2b–2n.

Figure 2 presents the stepwise demagnetization of the individual Fe_3_O_4_ particle shown in Figure 1. The BF TEM image of Figure 2a again displays the Fe_3_O_4_ particle, while Figures 2b–2n portray the response of its room temperature SIRM to stepwise heating and cooling. The initial magnetic‐induction map acquired at 20°C (Figure 2b) takes the form of a large counterclockwise spiraling vortex with an external stray magnetic field. Figures 2c–2n demonstrate the effect of stepwise heating and cooling of the Fe_3_O_4_ particle to increasing 100°C temperature intervals and back to 20°C after each heating step. Broadening of the phase contours for the Fe_3_O_4_ particle from 400°C is suggestive of a decrease in its magnetization strength with increasing temperature, until it is seen to be fully demagnetized at 600°C. Both the intensity and direction of the vortex PSD state are observed to be recovered when cooling back to 20°C (Figure 2n), similar to Figure 2b. The plot of Figure 2o displays the heating profile of the Fe_3_O_4_ particle in relation to magnetic‐induction maps of Figures 2b–2n.

**Figure 3:**
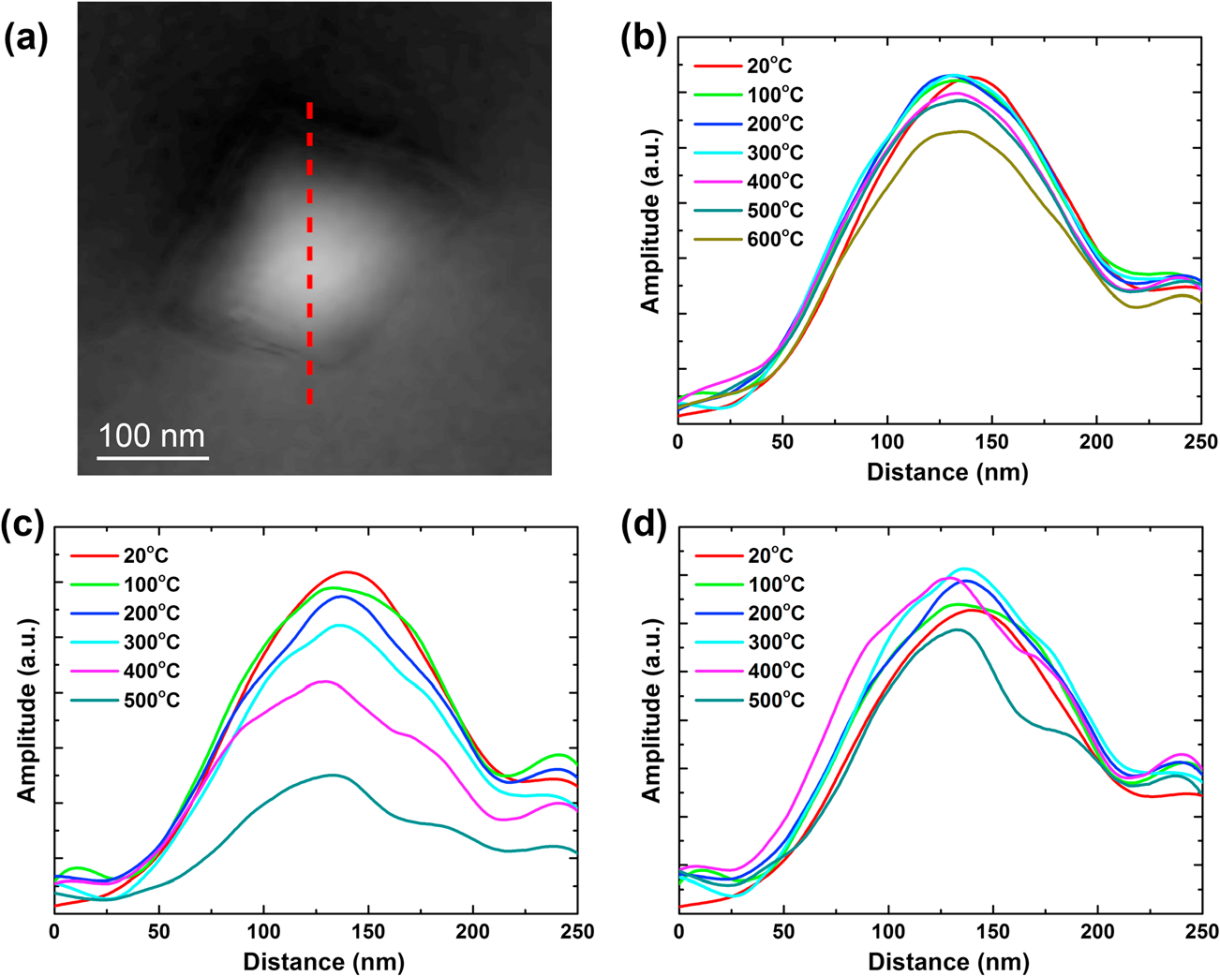
Magnetic additions to the phase shift. (a) An example of the magnetic addition to the phase image (acquired at 20°C) and (b) line profiles across the center of the Fe_3_O_4_ particle in the phase image (red line in Figure 3a) obtained at 20°C, after heating to their respective temperature intervals. Plots showing the line profiles acquired at (c) each temperature interval and (d) after correction for the temperature dependence of the spontaneous magnetization [*Dunlop and Özdemir*, 1997].

Using the magnetic‐induction maps in Figure 2, we analyzed the phase images (Figure 3a) and took line profiles across the center of the particle at various stages of the stepwise heating (Figures 3b and 3c). Figure 3b shows the line profiles of the magnetic addition that are taken at 20°C after heating to their respective temperature intervals. The line profiles plotted in Figure 3c present the magnetic addition acquired at each temperature interval of heating, while Figure 3d includes the correction for the temperature dependence of spontaneous magnetization experienced by Fe_3_O_4_ [*Dunlop and Özdemir*, 1997].

**Figure 4:**
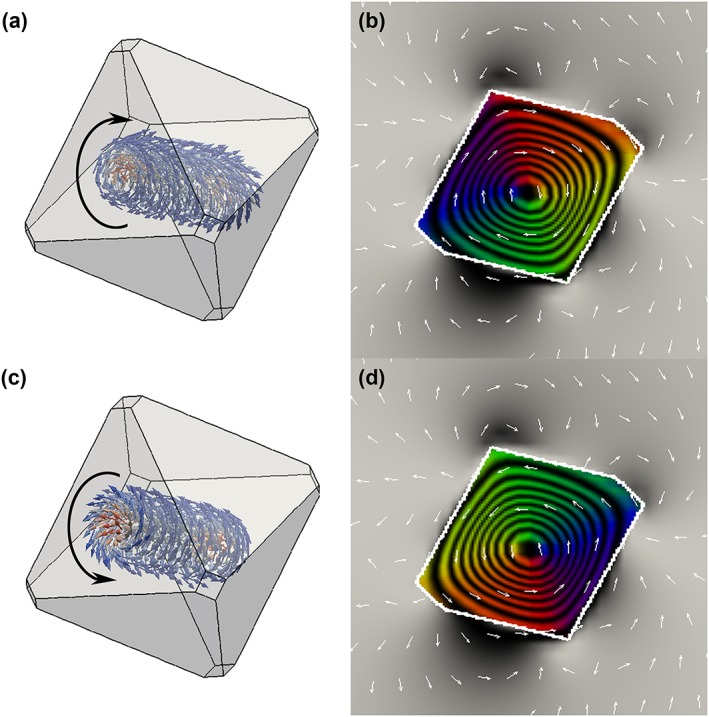
Computer simulations of the vortex structure held by the Fe_3_O_4_ particle. Micromagnetic modeling of the Fe_3_O_4_ particle showing magnetic moments flowing (a) clockwise or (c) counterclockwise around a vortex core and the (b and d) corresponding simulated magnetic‐induction maps.

The micromagnetic model of Figure 4a provides supporting information in the form of a 3‐D illustration of the magnetic domain structure of the Fe_3_O_4_ particle presented in Figure 2; the particle contains a clockwise vortex core aligned along a <111> direction. From the micromagnetic model in Figure 4a, we simulated a magnetic‐induction map image (Figure 4b). These evenly spaced contours flow in a clockwise direction (signified by arrows), and the image compares well with the experimental induction map (Figure 2b). Likewise, the micromagnetic model of Figure 4c and simulated magnetic‐induction map of Figure 4d show a similar relationship but with vorticity flowing in the counterclockwise direction.

## 4 Discussion

This study has provided structural and thermomagnetic characterization of the PSD grains, as well as direct visual insight into the stability and thermal demagnetization of an individual PSD Fe_3_O_4_ particle. The Mössbauer data have exhibited sextets with hyperfine parameters indicative of an approximate composition of 59 wt% Fe_3_O_4_ and 41 wt% γ‐Fe_2_O_3_, suggesting that the bulk powder is nonstoichiometric Fe_3_O_4_. The slightly oxidized particles are considered to adopt a Fe_3_O_4_/γ‐Fe_2_O_3_ core‐shell structure with a graded phase transition across the core‐shell interface, as described previously [*Almeida et al*., 2015]. The γ‐Fe_2_O_3_ shell is not considered to have a significant effect on the behavior of the vortex state in the relatively large Fe_3_O_4_ particle (~250 nm), other than to reduce slightly its vorticity [*Ge et al*., 2014]. Conventional thermal demagnetization measurements show that the magnetic signal held by the Fe_3_O_4_ grains is relatively stable up to ~500°C, above which the stability decreases rapidly on heating close to their *T_C_*, previously measured as 585 ± 5°C [*Almeida et al*., 2014c]. The magnetic‐induction map of the ~250 nm Fe_3_O_4_ particle (Figure 1d) clearly shows that the room temperature SIRM is a vortex. The corresponding simulated magnetic‐induction map (Figure 4b) closely matches the experimental induction map, confirming the micromagnetic vortex solution; the Fe_3_O_4_ particle contains a suitable PSD/vortex structure for studying the thermal response of nonideal magnetic recorders. The magnetic intensity of the vortex state decreases on heating to 500°C and fully demagnetizes at 600°C, but is recovered upon cooling with a reversal in vorticity, shown in the micromagnetic model and holography simulation of Figures 4c and 4d. Hence, the vortex state is observed to be thermally stable close to the *T_C_* and recovered after demagnetization, albeit with reversed vorticity. The spontaneous recovery of the vortex core along the same axis is considered to be influenced slightly by a combination of shape anisotropy and the weak ambient field of <0.2 mT. The magnetic signal recorded by this vortex state can therefore, in this instance, assume four possible variations: (1) clockwise or (2) counterclockwise vorticity, with the direction of the vortex core axis pointing (3) upward or (4) downward, out of plane. Electron holography is limited to the in‐plane magnetic component, and hence, the direction of the vortex core is unknown. Nevertheless, since the paleodirectional information is recovered from the direction along the vortex‐core axis, which can assume only one of two possible orientations, the particle can thus be said to behave like a uniaxial SD particle at temperatures approaching the *T_C_*.

The stepwise thermal demagnetization experiment (Figure 2) provides insight into the demagnetization response of the vortex structure, where variations in the magnetic contour width demonstrate changes in remanent intensity. This relationship is seen more clearly in the line profiles (Figures 3b–3d), and Figure 3b shows that the amplitude of the remanent intensity is generally fully recovered after heating to 100–500°C, and only after demagnetization at 600°C a 15–20% reduction of recovered intensity is observed, considered due to slight realignment of the vortex core. Considering that the direction of the particle's moment can change direction on heating to 600°C, then a measurement of zero net magnetization from a distribution of such particles, as often found in rocks, will suggest that they have demagnetized. However, the demagnetization is due to the random realignment of the grains' moments to cancel each other out and not due to their individual intensity. The line profiles of Figure 3c show a small decrease in amplitude of remanent intensity up to 300°C, which becomes more pronounced at 400°C, but the largest reduction can be seen at 500°C. However, when the correction for decrease in spontaneous magnetization with temperature is applied (Figure 3d), the amplitude in magnetic intensity becomes more comparable, implying that the vortex structure itself remained thermomagnetically stable. It is also apparent that the centers of the profiles become relatively more pronounced with increasing temperature, implying that the vortex core may be less sensitive to thermal effects and better preserve the magnetic intensity. One caveat of this experiment is that it was performed within 1 day and hence does not address the problem of TRM aging [*Shaar and Tauxe*, 2015]. Nevertheless, the particle is observed here to essentially behave like uniaxial SD recorders with a limited choice of direction of magnetic moments, suggesting that vortex states are reliable carriers for recovering ancient directional and intensity information.

## References

[grl54809-bib-0001] Almeida, T. P. , A. R. Muxworthy , W. Williams , T. Kasama , and R. E. Dunin‐Borkowski (2014a), Magnetic characterization of synthetic titanomagnetites: Quantifying the recording fidelity of ideal synthetic analogs, Geochem. Geophys. Geosyst., 15, 161–175, doi:10.1002/2013GC005047.

[grl54809-bib-0002] Almeida, T. P. , T. Kasama , A. R. Muxworthy , W. Williams , L. Nagy , P. D. Brown , and R. E. Dunin‐Borkowski (2014b), Visualised effect of oxidation on magnetic recording fidelity in pseudo‐single‐domain magnetite particles, Nat. Comm., 5, 5154, doi:10.1038/ncomms6154. 10.1038/ncomms6154 PMC4214405 25300366

[grl54809-bib-0003] Almeida, T. P. , T. Kasama , A. R. Muxworthy , W. Williams , L. Nagy , and R. E. Dunin‐Borkowski (2014c), Observing thermomagnetic stability of nonideal magnetite particles: Good paleomagnetic recorders?, Geophys. Res. Lett., 41, 7041–7047, doi:10.1002/2014GL061432.

[grl54809-bib-0004] Almeida, T. P. , A. R. Muxworthy , T. Kasama , W. Williams , C. Damsgaard , C. Frandsen , T. J. Pennycook , and R. E. Dunin‐Borkowski (2015), Effect of maghemization on the magnetic properties of nonstoichiometric pseudo‐single‐domain magnetite particles, Geochem. Geophys. Geosyst., 16, 2969–2979, doi:10.1002/2015GC005858.

[grl54809-bib-0005] Almeida, T. P. , A. R. Muxworthy , A. Kovács , W. Williams , P. D. Brown , and R. E. Dunin‐Borkowski (2016), Direct visualization of the thermomagnetic behavior of pseudo‐single‐domain magnetite particles, Sci. Adv., 2 e1501801. 10.1126/sciadv.1501801 PMC4846437 27152353

[grl54809-bib-0006] Beleggia, M. , and G. Pozzi (2010), Phase shift of charged metallic nanoparticles, Ultramicroscopy, 110, 418–424.

[grl54809-bib-0007] Biggin, A. J. , S. Badejo , E. Hodgson , A. R. Muxworthy , J. Shaw , and M. J. Dekkers (2013), The effect of cooling rate on the intensity of thermoremanent magnetization (TRM) acquired by assemblages of pseudo‐single domain, multidomain and interacting single‐domain grains, Geophys. J. Int., 193, 1239–1249.

[grl54809-bib-0009] Chang, S. L. Y. , C. Dwyer , J. Barthel , C. B. Boothroyd , and R. E. Dunin‐Borkowski (2016), Performance of a direct detection camera for off‐axis electron holography, Ultramicroscopy, 161, 90–97. 26630072 10.1016/j.ultramic.2015.09.004

[grl54809-bib-0010] Da Costa, G. M. , C. Blanco‐Andujar , E. De Grave , and Q. A. Pankhurst (2014), Magnetic nanoparticles for in vivo use: A critical assessment of their composition, J. Phys. Chem. B, 118, 11,738–11,746. 10.1021/jp5055765 25211599

[grl54809-bib-0011] Dunin‐Borkowski, R. E. , M. R. McCartney , R. B. Frankel , D. A. Bazylinski , M. Pósfai , and P. R. Buseck (1998), Magnetic microstructure of magnetotactic bacteria by electron holography, Science, 282, 1868–1870. 9836632 10.1126/science.282.5395.1868

[grl54809-bib-0012] Dunlop, D. J. , and Ö. Özdemir (1997), Rock magnetism: Fundamentals and frontiers, 577 pp., Cambridge Univ. Press, New York.

[grl54809-bib-0013] Dunlop, D. J. , and K. S. Argyle (1991), Separating multidomain and single‐domain‐like remanences in pseudo‐single‐domain magnetites (215–540 nm) by low‐temperature demagnetisation, J. Geophys. Res., 96, 2007–2017, doi:10.1029/90JB02338.

[grl54809-bib-0014] Ge, K. , W. Williams , Q. Liu , and Y. Yu (2014), Effects of the core‐shell structure on the magnetic properties of partially oxidized magnetite grains: Experimental and micromagnetic investigations, Geochem. Geophys. Geosyst., 15, 2021–2038, doi:10.1002/2014GC005265.

[grl54809-bib-0015] Harrison, R. J. , R. E. Dunin‐Borkowski , and A. Putnis (2002), Direct imaging of nanoscale magnetic interactions in minerals, Proc. Nat. Am. Soc., 99, 16,556–16,561. 10.1073/pnas.262514499 PMC139182 12482930

[grl54809-bib-0016] Kamilov, I. K. , G. M. Shakhshaev , K. K. Aliev , G. G. Musaev , and M. M. Khamidov (1975), Some features of the behavior of the thermal conductivity of ferrites in the vicinity of magnetic phase transitions, JETP, 41(2), 290–296.

[grl54809-bib-0017] Kasama, T. , R. J. Harrison , N. S. Church , M. Nagao , J. M. Feinberg , and R. E. Dunin‐Borkowski (2013), Ferrimagnetic/ferroelastic domain interactions in magnetite below the Verwey transition. Part I: electron holography and Lorentz microscopy, Phase Transitions, 86, 67–87.

[grl54809-bib-0018] Keimpema, K. , H. De Raedt , and J. T. M. De Hosson (2006), Electron holography image simulation of nanoparticles, J. Comp. Theor. Nano., 3, 362–374.

[grl54809-bib-0019] Manahan, M. P. (1990), Thermal expansion and conductivity of magnetite flakes taken from the Oconee‐2 steam generator, J. Mater. Sci., 25, 3424–3428.

[grl54809-bib-0020] Muxworthy, A. R. , D. J. Dunlop , and W. Williams (2003), High‐temperature magnetic stability of small magnetite particles, J. Geophys. Res., 108(B5), 2281, doi:10.1029/2002JB002195.

[grl54809-bib-0021] Néel, L. (1949), Théorie du trainage magnétique des ferromagnétiques en grains fins avec application aux terres cuites, Ann. Géophys., 5, 99–136.

[grl54809-bib-0022] Néel, L. (1955), Some theoretical aspects of rock magnetism, Adv. Physiol. Educ., 4, 191–242.

[grl54809-bib-0023] Shaar, R. , and L. Tauxe (2015), Instability of thermoremanence and the problem of estimating the ancient geomagnetic field strength from non‐single‐domain recorders, Proc. Natl. Acad. Sci. U.S.A., 112, 11,187–11,192. 10.1073/pnas.1507986112 PMC4568663 26305946

[grl54809-bib-0024] Thomson, L. C. , R. J. Enkin , and W. Williams (1994), Simulated annealing of 3‐dimensional micromagnetic structures and simulated thermoremanent magnetization, J. Geophys. Res., 99, 603–609, doi:10.1029/93JB02638.

[grl54809-bib-0025] Winklhofer, M. , K. Fabian , and F. Heider (1997), Magnetic blocking temperatures of magnetite calculated with a three‐dimensional micromagnetic model, J. Geophys. Res., 102, 22,695–22,709, doi:10.1029/97JB01730.

